# Different Serologic Behavior of MCPyV, TSPyV, HPyV6, HPyV7 and HPyV9 Polyomaviruses Found on the Skin

**DOI:** 10.1371/journal.pone.0081078

**Published:** 2013-11-21

**Authors:** Els van der Meijden, Seweryn Bialasiewicz, Rebecca J. Rockett, Sarah J. Tozer, Theo P. Sloots, Mariet C. W. Feltkamp

**Affiliations:** 1 Department of Medical Microbiology, Leiden University Medical Center, Leiden, The Netherlands; 2 Queensland Paediatric Infectious Diseases Laboratory, Queensland Children's Medical Research Institute, The University of Queensland, Brisbane, Australia; 3 Sir Albert Sakzewski Virus Research Centre, Royal Children's Hospital, Brisbane, Australia; Columbia University, United States of America

## Abstract

The polyomavirus family is rapidly expanding with twelve new human viruses identified since 2007. A significant number of the new human polyomaviruses (HPyV) has been found on the skin. Whether these viruses share biological properties and should be grouped together is unknown. Here we investigated the serological behavior of cutaneous HPyVs in a general population. 799 sera from immunocompetent Australian individuals aged between 0–87 were analyzed with a Luminex xMAP technology-based immunoassay for the presence of VP1-directed IgG antibodies against MCPyV, HPyV6, HPyV7, TSPyV, HPyV9, and BKPyV as a control. Except for HPyV9, overall seropositivity was high for the cutanous polyomaviruses (66–81% in adults), and gradually increased with age. Children below 6 months displayed seropositivity rates comparable to the adults, indicative of maternal antibodies. TSPyV seroreactivity levels strongly increased after age 2 and waned later in life comparable to BKPyV, whereas MCPyV, HPyV6 and HPyV7 seroreactivity remained rather stable throughout. Based on the identified serologic profiles, MCPyV seems to cluster with HPyV6 and HPyV7, and TSPyV and HPyV9 by themselves. These profiles indicate heterogeneity among cutaneous polyomaviruses and probably reflect differences in exposure and pathogenic behavior of these viruses.

## Introduction

The *Polyomaviridae* constitute a family of small DNA viruses that infect a variety of hosts. In recent years twelve new human polyomaviruses (HPyVs) were identified, among which the KI and WU polyomaviruses [1], [2], the Merkel cell polyomavirus (MCPyV) [3], the human polyomaviruses 6, 7, 9, 10 and 12 (HPyV6, HPyV7, HPyV9, HPyV10, HPyV12) [4]–[8], the trichodysplasia spinulosa associated polyomavirus (TSPyV) [9], the Malawi polyomavirus (MWPyV) [10], the Mexico polyomavirus (MXPyV) [11], and the STL polyomavirus (STLPyV) [12]. MWPyV, HPyV10 and MXPyV seem to belong to one species, because their whole genomes exceed the 81% identity criterion for polyomavirus species as proposed by the *Polyomaviridae* Study Group of the International Committee on Taxonomy of Viruses [13].

A number of the new HPyVs has been found on the skin (e.g. MCPyV, HPyV6, HPyV7, TSPyV, and HPyV9), with frequencies of 40–80% for MCPyV [4], [14], [15], 14–50% for HPyV6 [4], [16], 11% for HPyV7 [4], 2–4% for TSPyV [9], [17] and 1–17% for HPyV9 [5], [16]. In most healthy individuals, the viral DNA load on the skin was low. HPyV10 might also represent a cutaneous polyomavirus, as it was identified in an anal wart. However, the close resemblance with MWPyV, MXPyV, and the phylogenetically slightly more distant STLPyV, all found in feces, may suggest HPyV10 to be a fecal contaminant rather than a wart-causing skin virus.

Two of the cutaneous HPyVs, MCPyV and TSPyV, are considered to be pathogenic, and have stong evidence of causality in Merkel cell carcinoma (MCC) and trichodysplasia spinulosa (TS), respectively. MCPyV is clonally integrated in 80% of MCC, a rare but aggressive neuroendocrine skin tumor most commonly found in elderly and immunosuppressed persons [3], [18]. Recent improvements to the sensitivity of detection methods suggested that all MCCs harbor MCPyV [19]. Involvement of MCPyV in the pathogenesis of MCC is supported by clonal integration of MCPyV in MCC [3], the presence of specific MCPyV mutations that prevent viral replication while transformational property remains [20], [21], and the induction of elevated T antigen seroresponses in MCC patients [22]. Trichodysplasia spinulosa (TS) is a rare follicular skin disease exclusively found in severely immunocompromized hosts, characterized by papules and spicules (spines) mainly present on the face. Involvement of TSPyV in the pathogenesis of TS is supported by high TSPyV DNA detection rate and load in TS lesional vs. normal skin [9], [17], and abundant presence of VP1 protein and virus particles in affected follicular cells [17]. So far, HPyV6, HPyV7 and HPyV9 have not been associated with any disease.

Seroepidemiologic studies of the BK (BKPyV) and JC polyomaviruses (JCPyV) have indicated that these viruses are ubiquitous and infect the general population early in life with an overall seroprevalence of 60–80%, respectively [23]. For MCPyV, seroprevalences of 40–80% were reported in general immunocompetent populations [4], [23]–[27], and 70% for TSPyV [27]–[29]. For HPyV6 and HPyV7, seroprevalences of 70–85% and 35–60% were reported in healthy individuals, respectively [4], [27]. Lower (30–50%) seroprevalence rates in immunocompetent populations were shown for HPyV9 [27], [30], [31]. Of note, seroprevalences deduced from seroresponses directed against VP1 expressed as either virus-like particle (VLP) or GST-fusion protein, were generally comparable.

In this study we systematically determined and compared seropositivity and seroreactivity rates of the pathogenic cutaneous polyomaviruses MCPyV and TSPyV with those of HPyV6, HPyV7 and HPyV9 so far without attributable disease. To that purpose, we extended our previously described Luminex xMAP technology-based TSPyV VP1 polyomavirus seroassay [29] with VP1 antigen of MCPyV, HPyV6, HPyV7 and HPyV9. Age-specific seroresponses were determined for these five cutaneous HPyVs, in a group of 799 immunocompetent individuals from Queensland, Australia. BKPyV VP1 was included as a positive control antigen. Based on the observed differences and similarities in seropositivity and seroreactivity rates, the cutaneous HPyVs were grouped in three different serological profiles. These profiles were mutually compared and discussed with respect to known aspects of viral infection and pathogenicity.

## Materials and Methods

### Study population

For the purposes of this study, sera from a group of 799 immunocompetent Australian individuals were assessed. These samples were collected during October 2008 – June 2009 as part of a larger study investigating the seroprevalence of Q Fever in Queensland, Australia [32]. The study population consisted of three collection sites across the South East Queensland region, which is the most densely populated area of the state and includes the capital city of Brisbane. The principal sample set (n = 503) was obtained from serum collected for routine diagnostic testing at the largest tertiary public hospital in Brisbane and Queensland, the Royal Brisbane & Women's Hospital.

Additional sera (n = 110) collected for routine diagnostic testing were obtained from the public Toowoomba Hospital, which services the regional city of Toowoomba and the surrounding rural population. Sera from both hospitals were originally collected for a wide range of routine serological and biochemical testing. Clinical records were used, when available, to exclude immunocompromized patient sera from the candidate sample pool. A third sample group (n = 186) was obtained from children's sera submitted for routine allergy screening at a Brisbane-based private pathology clinic.

The age range of the total study population was 0–87 years (mean 25 yrs, median 15 yrs), with an overrepresentation of ages from 0–10 yrs. For sub-group analyses to determine seroprevalence by age, the total population was divided in 14 different age groups: 0–0.5 years (n = 31), 0.6–1.9 (n = 63), 2–3 (n = 62), 4–5 (n = 58), 6–7 (n = 58), 8–9 (n = 70), 10–14 (n = 55), 15–19 (n = 59), 20–29 (n = 59), 30–39 (n = 64), 40–49 (n = 54), 50–59 (n = 58), 60–69 (n = 54), and >70 years of age (n = 54) (**Table S1**). Appropriate approval for this research was obtained from the Queensland Children's Health Services Ethics Committee. Individual patient consent was not sought due to the retrospective nature of the study. The data were analyzed anonymously.

### Cloning and expression of GST-VP1.tag fusion proteins

In the polyomavirus multiplex serology methodology, Glutathione-S-Transferase (GST)-VP1.tag fusion proteins were used as antigen [33]. The construction and expression of GST-MCPyV344 VP1.tag, GST-TSPyV VP1.tag and GST-BKPyV VP1.tag was performed as described [29]. To obtain the pGEX-HPyV6 VP1.tag construct, the VP1 gene of HPyV6 was cloned into the pGEX5x3 vector (Amersham Biosciences). In the PCR reaction, p6VP1 vector (Addgene plasmid 24725) [4] was used as input combined with the following primers with restriction site linkers (underlined) and a tag sequence (italic) at the 3′-end: HPyV6 sense 5′-GAATTCGAATTCTCCCTGCCATCGCAAAGGCAACG-3′, HPyV6 anti sense 5′- GCGGCCGCT*GTTTCAGGTTCAGGGGGAGGTGTGGGAGGTTT*AAGCTTGAGTTCGCCCTTGCTGGGCTCCTT-3′. For pGEX-HPyV7 VP1.tag, the HPyV7 VP1 gene was cloned into the pGEX4t3-MCPyV VP1.tag vector after removal of the MCPyV VP1 sequence. For this purpose, pHPyV7-713a vector (Addgene plasmid 24728) [4] was used as input for the PCR reaction with the following primers with restriction site linkers (underlined): HPyV7 sense 5′- GGATCCGGATCCCCCTGTCAAAGAAAAGGAAATGG-3′ and HPyV7 anti sense 5′-GTCGACGTCGACAATTCCTTCTTGTTGTCTGTG-3′. For PCR and cloning strategy details see van der Meijden et al. [29]. A synthetic clone of pGEX5x3-HPyV9 VP1.tag was constructed by GenScript based on the HPyV9 VP1 sequence with RefSeq NC_015150. GST-HPyV VP1.tag fusion proteins were expressed in the Bl21 Rosetta *Escherichia coli* strain as described [24], [33]. Comparable expression of all GST-VP1 fusion proteins was checked by western blotting using either an anti-tag antibody (1∶5000 dilution, a generous gift from Waterboer and Pawlita) [34] or an anti-GST antibody (1∶5000 dilution, Santa Cruz) (data not shown).

### Multiplex polyomavirus serology

For the measurement of antibodies against the HPyVs described above, a multiplex antibody binding assay was performed (Luminex based xMAP technology). This assay is based on fluorescent beads chemically cross-linked to glutathione-casein (GC) to which, through affinity purification of 1 mg/ml crude bacterial GST-VP1.tag fusion protein lysate, the GST-VP1.tag fusion proteins could bind [33]. The quality of GST-VP1.tag binding to the beads was tested by detection with either an α-tag or α-GST antibody. This resulted in approximately comparable GST-HPyV VP1.tag bead binding. Multiplex serology was performed as described in van der Meijden et al. [29]. In short, the serum samples were tested in a 1∶100 dilution in blocking buffer (1 mg/ml casein (Sigma), 0.5% polyvinylalcohol (Pierce), 0.8% polyvinylpyrrolidone (Pierce), 2.5% Super ChemiBlock (CBS-K, Chemicon international) in PBS) incubated for 1 hour at room temperature to suppress non-specific binding of antibodies to the beads. Furthermore, the blocking buffer also contained 2 mg/ml lysate from bacteria expressing GST.tag alone, to block antibodies directed against residual bacterial proteins and GST.tag. For competition experiments serial serum dilutions were either pre-incubated with lysate from bacteria expressing GST.tag alone or with the individual GST-VP1.tag fusion protein lysate. Subsequently, serum samples were incubated for 1 hour at room temperature with the GST-VP1.tag coupled beads. For the detection of VP1-bound antibodies, biotinylated goat-anti-human IgG (H+L) (1∶1000, Jackson ImmunoResearch Laboratories Inc.) followed by streptavidine-R-phycoerythrin (1∶1000, Invitrogen) were used, each with an incubation step of 30 minutes at room temperature. Finally, the beads and the phycoerythrin signal were analyzed in a Bio-Plex 100 analyzer (Bio-Rad Laboratories) resulting in median fluorescent intensity (MFI) units. To correct for background seroreactivity, MFI values measured against GST alone were subtracted to obtain HPyV VP1 specific signals. On every plate a serum pool of 4 serum samples was included as reference for the multiplex serology. For this purpose 1∶4 serial dilutions of the serum pool were made starting with a dilution of 1∶100 up to 1∶409.600 and the seroresponse against MCPyV, HPyV6, HPyV7, TSPyV, HPyV9, and BKPyV was measured. Little variance was observed between the plates.

### Cut-off value determination

Immunoassay cut-off values were based on the group of immunocompetent children aged 7–24 months (n = 63) among the study population. A frequency distribution analysis with a bin width of 250 MFI was performed on the seroresponses of the cut-off population for each HPyV tested, to determine the seronegative population, which was defined by samples falling within bins with a frequency percentage above 10% (**Figure S1, red bars**). Cut-off values were calculated by the mean seroresponse for each virus of the serononresponders plus three times the standard deviation. While doing so, the following cut-off values were obtained: MCPyV, 321 MFI; HPyV6, 129 MFI; HPyV7, 604 MFI; TSPyV, 250 MFI; HPyV9, 317 MFI; BKPyV, 428 MFI.

### Statistical analysis

**Figure 1:**
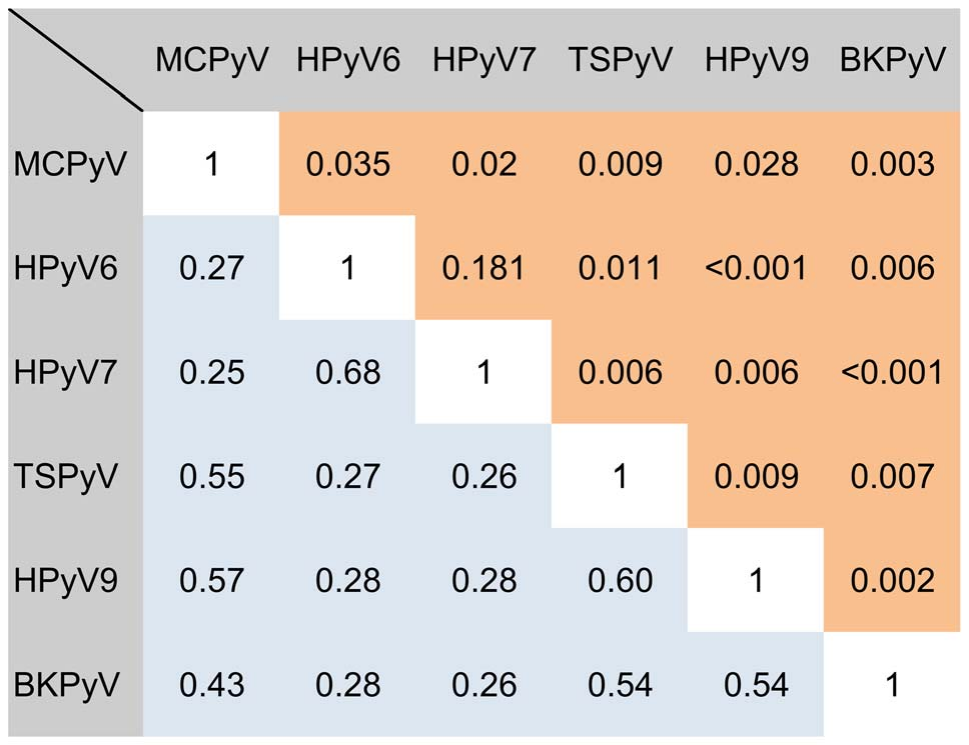
VP1 sequence identities and seroresponse correlations between tested polyomaviruses. The lower-left triangle shows homology (percentage sequence identity; 1 corresponds to 100% identity) between VP1 protein sequences of the six polyomaviruses tested generated by ClustalW alignment using Geneious Pro 5.6.3 software with default settings. For this alignment the following RefSeq/GenBank accession numbers were used: MCPyV, NC_010227; HPyV6, NC_014406; HPyV7, NC_014407; TSPyV, NC_014361; HPyV9, NC_015150; BKPyV, NC_001538. The upper-right triangle shows cross-reactivity between seroresponses measured against VP1 of the six polyomaviruses tested. For this analysis the serologic data from the complete study population were used (n = 799). Pearson correlation coefficients (r^2^) were determined by SPSS 20 software (IBM).

Pearson correlation coefficients (r^2^) were calculated to determine the association between seroresponses against different polyomaviruses, as shown in **Figure 1** (upper-right triangle). Odds ratios (OR) were established to describe the association between seropositivity and age for each of the tested HPyVs, with age group 0.6–1.9 years as a reference. Differences in mean seroreactivity between the different age groups and age group 0.6–1.9 years (reference) were calculated with the Student's T-test. All statistical calculations were performed by using SPSS 20 statistical software (IBM).

## Results

### Seroresponses

**Figure 2:**
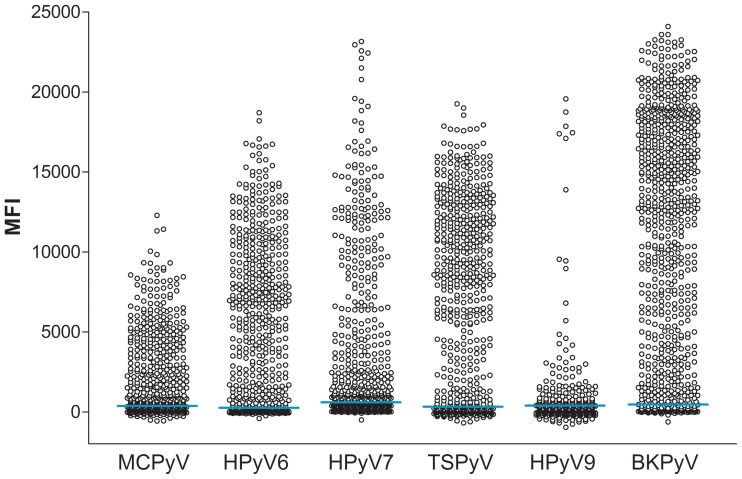
Seroresponses measured against VP1 of MCPyV, HPyV6, HPyV7, TSPyV, HPyV9 and BKPyV. Presented are seroresponses of 799 Australian immunocompetent individuals. Each circle corresponds to the mean fluorecence intensity (MFI) measured for an individual serum sample in the Bio-Plex 100 analyzer. The cut-off value calculated for each polyomavirus as explained in **Figure S1** is depicted as a blue line.

To measure seroresponses against MCPyV, HPyV6, HPyV7, TSPyV, HPyV9 and BKPyV, the VP1 major capsid protein of each polyomavirus was expressed and coupled to specifically colored Luminex beads. Seroresponses against the VP1-coated antigenic beads were measured for each individual within the study population using the Luminex xMAP technology. The obtained serological measurements are shown separately for each polyomavirus in **Figure 2** (**Dataset S1**, Excel file raw data). Seroresponses were common for TSPyV, HPyV6 and HPyV7, intermediate for MCPyV, and low for HPyV9. The highest seroresponses were measured for BKPyV.

### Sero-crossreactivity

Based on shared VP1 amino acid sequence identity (**Figure 1**, lower-left triangle), cross-seroreactivity might be expected between phylogenetically closely related polyomaviruses, such as HPyV6 and HPyV7 (68% identity) [13], [35]. To explore this potential drawback, for the whole study population a correlation analysis was performed between the seroresponses measured against each of the VP1 antigens. Poor or absent correlations were observed between seroresponses against the tested polyomaviruses, with that between HPyV6 and HPyV7 being the highest (r^2^ = 0.181) (**Figure 1**, upper-right triangle).

**Figure 3:**
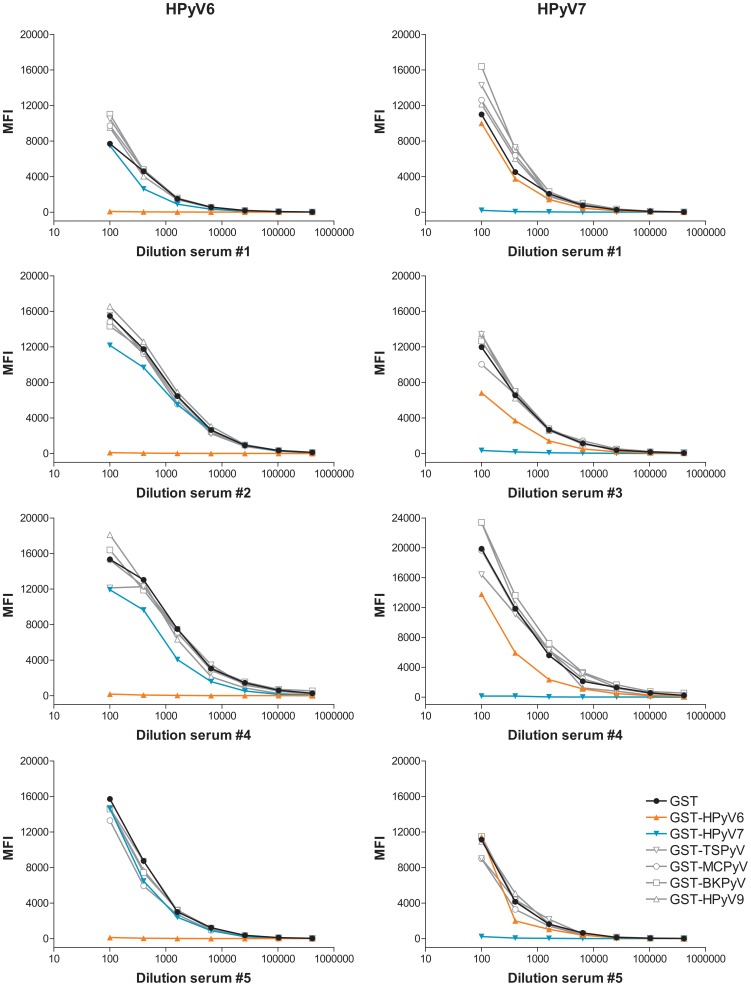
VP1-specific competition of polyomavirus seroresponses. Serial dilutions of four serum samples were preincubated with soluble recombinant fusion protein lysate containing GST.tag alone (black), GST-HPyV6 VP1.tag (orange), GST-HPyV7 VP1.tag (blue) or GST-TSPyV, GST-MCPyV, GST-BKPyV, GST-HPyV9 (grey). The HPyV6 (left part of the figure) and HPyV7 (right part of the figure) VP1 seroreactivity was measured by the Bio-Plex 100 analyzer. MFI stands for median fluorescent intensity.

VP1 antigen-competition experiments were performed to further exclude cross-reactivity between HPyV6 and HPyV7. Serial dilutions of four HPyV6 and HPyV7-reactive serum samples were preincubated separately with lysates from bacteria expressing the individual GST-VP1.tag fusion proteins or the GST.tag alone, after which HPyV6 and HPyV7 seroresponses were measured. In all cases, the HPyV6 and HPyV7-directed seroresponses were completely inhibited after pre-incubation with the homologous VP1 antigen (**Figure 3**), whereas pre-incubation with heterologous polyomavirus VP1 antigens did not affect the measured seroresponses against HPyV6 or HPyV7. In two sera tested however (#2 and #4 for HPyV7, and #3 and #4 for HPyV6; **Figure 3**), a subtle reduction in HPyV6 and HPyV7 seroreactivity was observed after pre-incubation with the reciprocal VP1 antigen, in line with the observed poor correlation calculated for the total serum set (**Figure 1**).

### Seropositivity

**Figure 4:**
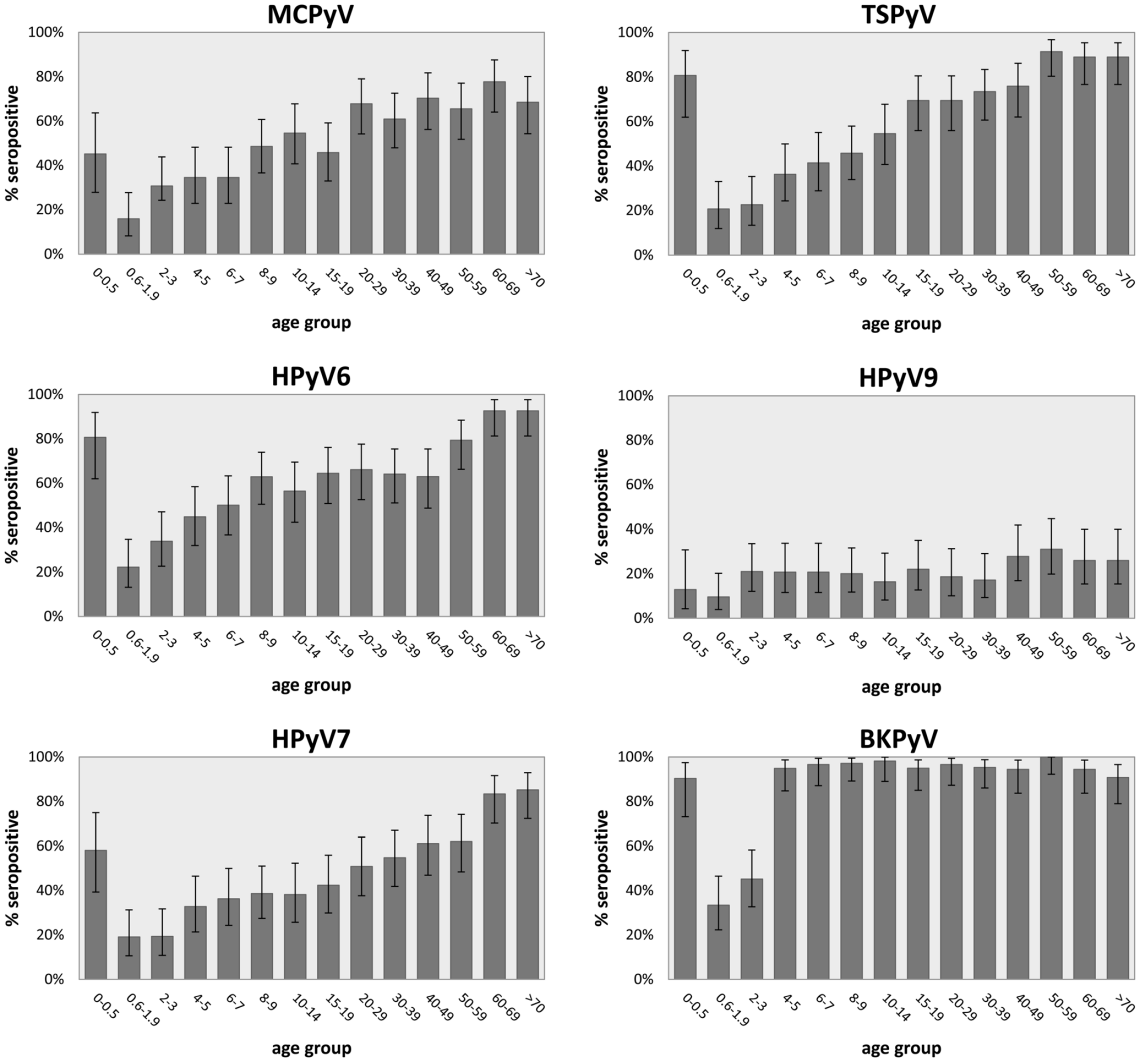
Seropositivity of MCPyV, HPyV6, HPyV7, TSPyV, HPyV9 and BKPyV. Seropositivity is measured for the total study population divided into 14 different age groups. Error bars indicate 95% confidence intervals.

To determine the seroprevalences in our study population for each of the viruses, seropositivity was assessed by calculating the proportion of sera that displayed seroreactivity above the established cut-off value for each virus (**Figure S1**). In **Figure 4**, the proportional seropositivity is shown for each polyomavirus, distributed by age group. A significant increase in seropositivity with age was observed for all viruses tested, although for HPyV9 significance was marginal and reached only in the older age groups (**Table S2**).

In the 0.6–1.9 year-of-age group, seropositivity varied between 10–20%. By adulthood (>20 years-of-age) seropositivity rates increased to 68% for MCPyV, 76% for HPyV6, 66% for HPyV7 and 81% for TSPyV. Adult seropositivity for HPyV9 remained low (24%). Only 4 adult samples (1.2%) were seronegative for any of the cutaneous HPyVs. Seropositivity for all of the tested polyomaviruses was observed in 56 individuals (7%) of which 43 (77%) were adults.

Except for HPyV9, in the age group below 6 months of age, seropositivity was significantly higher than in the next age group and comparable to the levels measured in the adults (**Figure 4** **and Table S2**). This phenomenon was also observed for BKPyV, where a seropositivity of 90% was measured in the very young, in line with very high seropositivity rates in the higher age groups.

### Intensity of seroresponses

**Figure 5:**
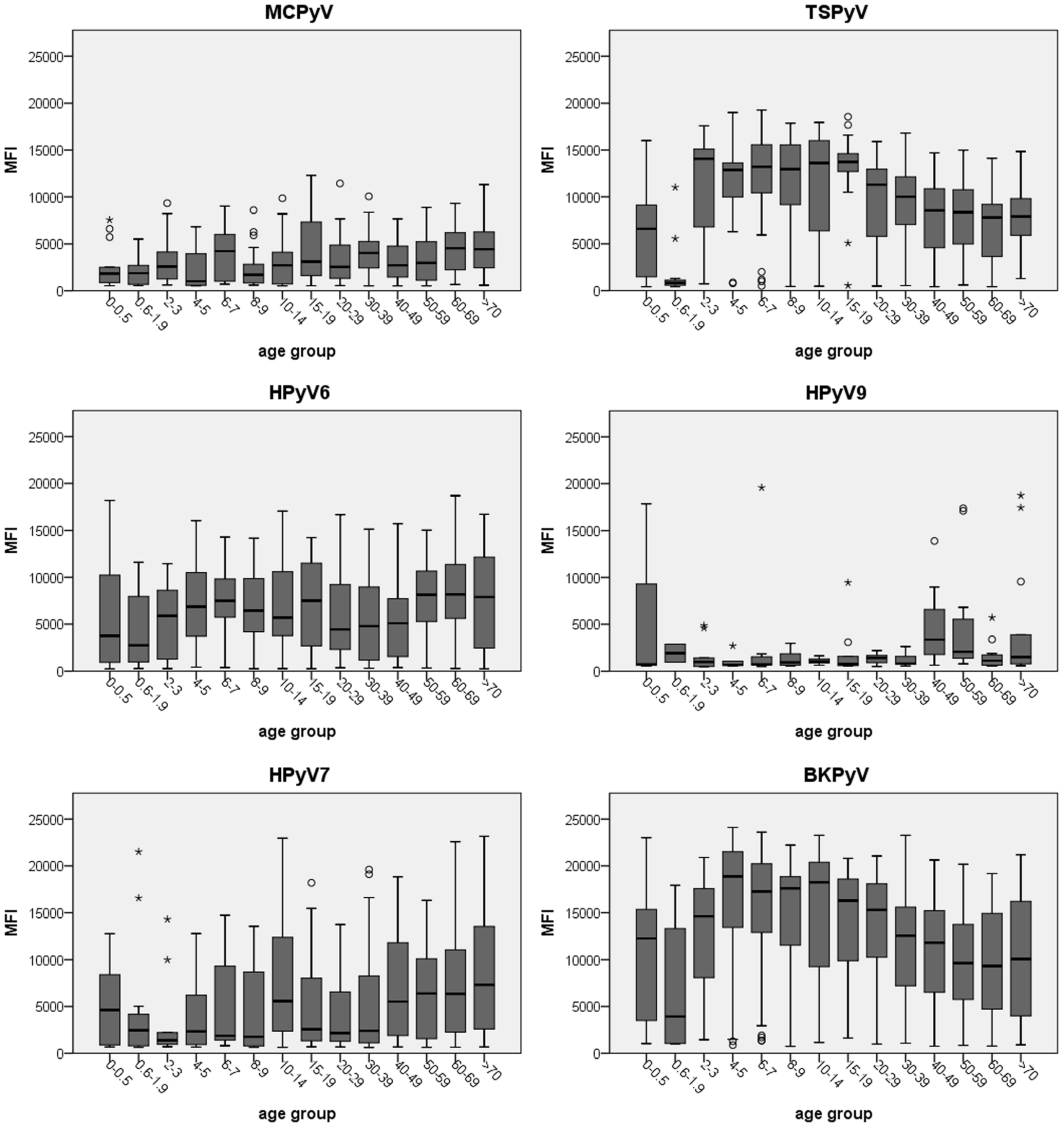
Age-specific seroreactivity against MCPyV, HPyV6, HPyV7, TSPyV, HPyV9 and BKPyV. The age-specific seroreactivity is measured amongst the respective seropositive individuals. The horizontal line in each boxplot represents the median MFI value. The box includes the 25%–75% MFI value range and the whiskers the 5%–95% MFI value range. Outliers are represented as a circle and extreme outliers as an asterisk.

Among the seropositive individuals, the intensity of the seroresponses was analyzed for each virus in the different age groups. From 0.6 years-of-age on, a strong increase in median seroreactivity was observed for TSPyV that peaked between 2 and 20 years-of-age (**Figure 5**), and decreased later in life to roughly half of the intensity of the peak level. The mean TSPyV seroreactivity was significantly increased in all age groups compared to the mean seroreactivity measured in age group 0.6–1.9 years of age (p<0.001, Student's T-test) (**Table S3**). This pattern mirrored to what was observed for BKPyV in the same analysis (**Figure 5**). The seroreactivity against MCPyV, HPyV6 and HPyV7 only slightly increased with age. For HPyV9 the number of seropositive individuals was too small to draw any conclusions in this regard.

## Discussion

In order to investigate seroresponses against HPyVs found on the skin, we extended our TSPyV VP1-antigen immunoassay with MCPyV, HPyV6, HPyV7 and HPyV9. Except for MCPyV, the intensities of the measured seroresponses were in general comparable for the tested HPyVs. Fusion protein expression and bead binding were comparable between the six HPyVs and cannot explain the lower maximum MFI value observed for MCPyV (data not shown). Lower immunogenicity of the used MCPyV strain 344 might explain this observation, although it contains aspartic acid (D) and arginine (R) at positions 288 and 316, respectively, important for proper folding of the MCPyV VP1 protein and epitope recognition [24]. MCPyV strain 344 has a VP1 sequence identical to strain 339 used in most MCPyV seroepidemiological investigations.

Minor cross-serorecognition was observed between HPyV6 and HPyV7 but this correlation was very poor, both in the overall comparison and in the competition experiments. Another recent study using VP1 VLP antigen also had some indication of cross-serorecognition of these viruses [27]. Taken together, the relevance of these findings for interpretation of our data was considered low, because both in our and in the French study the observed crossreactivity was weak, with a calculated Pearson correlation coefficient of 0.426, r^2^-value of 0.181 in our study, and a Spearman correlation coefficient of 0.433 in the French [27]. A study by Schowalter et al. [4] showed no crossreactivity between HPyV6 and HPyV7.

We cannot rule out the possibility of crossreactivity between the six HPyVs analyzed in this study and other polyomaviruses, especially ones with high VP1 homology [36], for instance as already described between HPyV9 and simian lymphotropic polyomavirus (LPyV) [30], [31]. However, several studies have demonstrated the absence of crossreactivity against the different HPyVs [23]–[25], even between polyomaviruses with high VP1 homology; e.g. BKPyV and JCPyV (78%) [23], [37], [38], and KIPyV and WUPyV (66%) [23], [39].

The observed seropositivity rates were within the range of previously published studies [4], [23]–[29], although the HPyV9 rate was somewhat lower [27], [30], [31]. This might be explained by lower circulation of HPyV9 in Queensland, Australia, compared to Europe and the USA [27], [30], [31], by possible local serotype variation or by different ways of exposure. An alternative explanation could be the use of the GST-VP1 based Luminex assay versus VP1 VLP based ELISA employed by others, although this seemed irrelevant for the other polyomaviruses tested. In general, seropositivity rates are influenced by the used cut-off values, which were defined arbitrarily and differed in the various studies.

The observed seropositivity patterns of MCPyV, HPyV6, HPyV7 and TSPyV indicate that primary infections commonly occur in children and young adults. The increase of seropositivity with age for most of the viruses tested is compatible with the model of continuing primary infections throughout adult life. High seropositivity rates were detected in the first 6 months of life for all viruses except HPyV9. The rapid decline to the lowest measured seropositivity rates for each virus in the consecutive (0.6–1.9 yrs) age group, strongly suggests the presence of maternal antibodies during infancy. Maternal antibodies against KIPyV and WUPyV in young infants have been previously reported by Nguyen et al. [39], and Boldorini et al. have shown a decrease over time of JCPyV and BKPyV IgG levels in follow-up samples of newborns [37].

Differences were observed with respect to the intensity of virus-specific seroreactivities among seropositive individuals. Whereas MCPyV, HPyV6 and HPyV7 seroreactivity moderately increased with age, the seroreactivity for TSPyV rapidly increased, analogous to BKPyV seroreactivity, as reported also by others [23], [40], [41]. Advancing adult age correlated with a gradual decrease of seroreactivity for both TSPyV and BKPyV. A similar serological TSPyV pattern was observed in a group of healthy individuals from the Netherlands (van der Meijden and Feltkamp, unpublished data), and in an Italian population recently described by Nicol et al. [27]. This pattern of waning seroresponses might be explained by immunosenescence or diminished boosting of seroreseponses by less virus exposure or infrequent reactivation.

Further analysis of our serological data revealed no associations between seropositivity and sex (data not shown). The association between HPyV9 seroprevalence and men described by Nicol et al. [30] was therefore not seen. We did observe some differences in seroresponses between individuals from the three collection centers, but all of these could be explained by mutual differences in age distribution (**Table S1**). No information was available on ethnicity, and specifically Australian Indigenous identity, so associations in this regard could not be investigated. Given the general demographics of the areas that were serviced by the three health centres, we expect the large majority of the study population to be Caucasian. Furthermore, we compared available clinical data of patients with either upper or lower quartile seroresponsiveness for each HPyV tested for conditions including upper or lower respiratory disease, gastroenteritis, organ dysfunction or reported cutaneous abnormalities, but no specific patterns or associations with disease were found.

Based on our data, three different serological profiles seem apparent for the cutaneous polyomaviruses. Profile 1 was exhibited by MCPyV, HPyV6 and HPyV7. It is characterized by a significant increase in seropositivity with age, while the intensity of the seroresponses remain rather stable. Profile 2 was observed for TSPyV. Comparable to Profile 1, Profile 2 is characterized by a significant increase in seropositivity with age, in this case combined with a steep seroreactivity increase during childhood that seems to wane later in life. Profile 3 observed for HPyV9 displayed low seropositivity throughout all ages, with occasionally high seroresponses.

The biological basis behind each polyomavirus' serological profile is unknown so far. In part they may reflect differences in virus exposure, porte d'entree and/or antigen presentation. Furthermore, the extent of infection within the host and immunogenicity of the virus possibly play a role. The close resemblance of Profile 2 (TSPyV) with that of BKPyV, for instance, could suggest that TSPyV causes a generalized primary infection with viremia. Consequently, this virus should not be considered a cutaneous pathogen *per se*. The low prevalence of TSPyV on the skin of healthy individuals (<5%) [9], [17], as well as detection of TSPyV DNA in the kidney [42] and lymph node tissue [8] may support this idea.

Conversely, it is tempting to speculate that serological Profile 1 (MCPyV, HPyV6 and HPyV7) indicates infection without viremia, for example limited to the skin. In light of the many reports that revealed the presence of DNA from these viruses in several different (internal) clinical samples, however, it is too early to draw any conclusions in this regard. The same is true for HPyV9 (Profile 3) that has been found both on skin and in blood [5], [6]. To what extent the reported detection rates of these ‘cutaneous’ viruses are skewed by viral DNA contamination from the skin of test subjects or sample handlers is unknown, but this may reflect a considerable risk of misinterpretation of data in these type of studies.

To summarize, on the basis of observed seropositivity and seroreactivity rates three different serologic profiles were discerned for the polyomaviruses found on the skin. These profiles probably reflect differences in exposure and/or in host and pathogenic behavior of these polyomaviruses, and further study in this regard is needed. Until that information is available, prudence is called to group these new viruses together as cutaneotropic.

## Supporting Information

Figure S1 **Frequency distribution of seroresponses.** Presented are seroresponses of children aged 0.6–1.9 years (n = 63) for MCPyV, HPyV6, HPyV7, TSPyV, HPyV9 and BKPyV. Bins with a width of 250 MFI were used and samples falling within bins with a frequency percentage > 10% were included for the cut-off value calculation (red bars).
(TIF)

Table S1 **Distribution of the serum samples over the three study centres of sample collection.**
(TIF)

Table S2 **Associations (odds ratios) between seropositivity and age.**
(TIF)

Table S3 **Median and mean seroreactivity among seropositive patients per age group.**
(TIF)

Dataset S1 **Dataset S1.**
(XLS)
